# Dose–Response Assay for Synthetic Mosquito (Diptera: Culicidae) Attractant Using a High-Throughput Screening System

**DOI:** 10.3390/insects12040355

**Published:** 2021-04-16

**Authors:** Dae-Yun Kim, Theerachart Leepasert, Michael J. Bangs, Theeraphap Chareonviriyaphap

**Affiliations:** 1 Department of Entomology, Faculty of Agriculture, Kasetsart Univeristy, Bangkok 10900, Thailand; daeyun.k@ku.th (D.-Y.K.); bangs_michael@yahoo.com (M.J.B.); 2 Department of Chemistry, Faculty of Science, Kasetsart University, Bangkok 10900, Thailand; jonnyj14@gmail.com

**Keywords:** yellow fever mosquito, *Aedes aegypti*, southern house mosquito, *Culex quinquefasciatus*, BG-lure^TM^, high-throughput screening system, olfactometer, attractant, lure, dose response

## Abstract

**Simple Summary:**

Entomological surveillance is important to evaluate vector management interventions. However, collecting adult mosquitoes using direct human bait is controversial and often discouraged because of potential infection risk. Alternatively, active and passive trapping methods are available. Female mosquitoes detect human host cues such as body heat, carbon dioxide, and other volatile body emanations using olfactory sensilla to direct movement to a host. Attractive chemical lures have been identified and evaluated using a variety of olfactometric methods to increase trap production and efficiency. In this study, we evaluated a simple olfactometer without need of airflow. To ‘optimize’ a commercial mosquito attractant, 10 different doses of product, the Biogents-lure (BG-lure^TM^), were compared. Results showed dose-dependent responses with 0.005 g with the highest attraction for *Aedes aegypti*, while doses of 0.2 g and above produced a repellent response. There was no significantly different response behavior between permethrin-susceptible and -resistant *Ae. aegypti*. *Culex quinquefasciatus* showed significantly different responses compared to *Ae. aegypti* by producing attraction over four times a wider range of amounts. These results demonstrate a simple olfactometer device to screen potential chemical attractants without use of an air-plume, thus expanding testing capabilities beyond more sophisticated laboratory settings.

**Abstract:**

Natural volatile host cues play a critical role for mosquito orientation and locating a blood source for egg production. Similar olfactory activation responses have allowed the use and development of artificial chemical attractants to lure mosquitoes to trapping devices. Using a pre-formulated commercial product mixture of different attractant chemicals, a high-throughput screening system (HITSS) is used to screen varying doses of chemical required to activate behavioral responses. Two strains of *Aedes aegypti* (L.): permethrin-susceptible (USDA) and -resistant (Pu Teuy) phenotypes and one *Culex quinquefasciatus* Say. (NIH) laboratory strain were tested. Overall, mosquitoes showed repellency between 1.0 g and to 10.0 g dose of each compound. However, by progressively reducing the dose, *Cx. quinquefasciatus* showed a greater positive percent attraction (88.9%) at 0.025 g, whereas the USDA and Pu Teuy *Ae. aegypti* produced optimum attractant activation at 0.005 g (72.6% and 58.9%, respectively) without significant difference within species (*p* > 0.05). In parallel control assays, *Cx. quinquefasciatus* was significantly attracted to 1 g of dry ice (carbon dioxide) (76%) more than *Ae. aegypti* (USDA) (12.2%). The HITSS was originally designed to measure three chemical actions to sublethal concentrations of chemicals by mosquitoes: toxicity and the two primary behavior avoidance responses (contact excitation and spatial repellency). These findings demonstrate that the HITSS assay, with only minor modifications, allows comparison screening of candidate compounds as potential attractants for anemotactic responses under laboratory-controlled conditions. Further investigations will be required to equate measurements obtained from controlled laboratory assays to more varied field conditions for attracting natural mosquito populations.

## 1. Introduction

The mosquitoes *Aedes aegypti* (L.) and *Culex quinqufasciatus* Say (Diptera: Culicidae) are species with a global reach and public health importance. For example, approximately half of the human population is at risk for infection with dengue viruses [1] transmitted primarily by *Aedes* mosquitoes (subgenus *Stegomyia*), particularly *Ae. aegypti*, a nearly globally distributed, eusynanthropic species that typically resides in and near human dwellings [2]. This species is also a primary vector of yellow fever, chikungunya, and zika viruses. *Cx. quinquefasciatus* is a cosmopolitan species throughout tropical and subtropical regions. Common in urbanized areas, it represents a primary pest species during evening hours. It is capable of transmitting *Wuchereria bancrofti* (lymphatic filariasis) and several virus pathogens to humans (e.g., West Nile and St. Louis Encephalitis) and animals [2].

Understanding female mosquito responses to human host is crucial for providing better comprehension of the epidemiology of pathogen transmission and applying preventative vector control measures. Diurnally active *Ae. aegypti* females seek blood primarily for reproductive purposes by using a complex set of sensory mechanisms directing host-seeking behavior [3,4]. Host seeking involves a series of in-flight orientation steps by an avid female toward a potential blood meal [5]. A sequential chain of actions includes attraction to host via ‘cues’ (orientation phase) and a series of additional steps after settling on the host, from probing, initiating blood feeding to engorgement, followed by withdrawal of the mouthparts from the host skin [3,6]. Each step is influenced by unique host stimuli detected by a variety of visual, mechanical, and chemical mosquito receptors [7]. In addition, host-seeking behavior is influenced by environmental factors such as ambient air temperature, relative humidity, and air movement.

Chemical cues derived from breath, skin, and excretions are present in the surrounding air column and used by hematophagous arthropods to detect hosts [8]. Mosquitoes perceive olfactory molecules via chemo-sensitive receptors in sensilla located on antennae and mouthparts (maxillary palps and labia) [9,10]. Female mosquitoes can detect hosts from varying distances along natural convection currents carrying airborne host emanations, in particular long-distance detection of carbon dioxide [11,12]. At closer distance, along with the host’s body heat (infrared spectrum) and surface moisture, various types of chemical categories such as short-chain carboxylic acids and aldehydes attract the female mosquitoes. Specific chemicals have been analyzed for their efficacy in blends rather than as a single compound. l-Lactic acid, ammonia, octenol (1-octen-3-ol), indole, nonanal (nonanaldehyde), and amino acids from red blood cells are the main molecules associated with body sweat and odors [13,14,15,16].

Host-derived chemicals and emanations, alone or in combination, are important signals (kairomones) for host-seeking responses by female mosquitoes [7,17,18]. For example, 2-butenone can induce and activate the neuronal receptors on a mosquito’s maxillary palp to detect acetone and cyclopentanone, which play key roles in the host-seeking process [19]. Investigations of odor-mediated host-seeking behavior require knowledge of the specific chemical components of complex host odors that act as powerful attractants and the concentration of odorant that contribute to the composite behavior of host seeking [20,21].

This study used a high-throughput screening system to measure varying dose responses of *Ae. aegypti* and *Cx. quinquefasciatus* to a commercial attractant product under laboratory-controlled conditions. By optimizing the dose of a compound to the specific assay conditions and design, the dosage can be used to compare response within and between adult insect species (e.g., mosquito) and serve as a valuable investigative tool to observe behavioral effects of numerous bioactive volatile compounds.

## 2. Materials and Methods

### 2.1. Mosquitoes

*Aedes aegypti* laboratory strain was obtained from the United States (US) Department of Agriculture (USDA), Gainesville, Florida, USA (ca. 1996), a colony continuously maintained under laboratory-controlled conditions for over 50 years and completely susceptible to insecticides [22]. *Culex quinquefasciatus* was obtained from the National Institute of Health (NIH), Department of Medical Sciences, Ministry of Public Health, Nonthaburi, Thailand, in 2015. This colony has been continuously maintained by the NIH for nearly 40 years. Lastly, a field population (Pu Tuey) of immature *Ae. aegypti* was collected in January 2019 from artificial containers near household at Pu Teuy Village in Kanchanaburi Province (14°17′ N, 99°11′ E), western Thailand. Larvae and pupae were immediately transferred to the Department of Entomology, Faculty of Agriculture, Kasetsart University in Bangkok, Thailand for initial rearing and colonization.

Immature stages were reared to adults under insectary-controlled conditions (25 °C ± 5 °C, 80% ± 10% RH, with a 12 h/12 h light/dark photoperiod). Adult mosquitoes were provided with cotton pads soaked with 10% sugar solution on first day of emergence with each strain maintained in separate rooms. The naturally inseminated female mosquitoes were permitted to feed on blood through an artificial membrane feeding system at day 3 post emergence. For *Ae. aegypti*, 2 days after blood-feeding, 10 cm diameter oviposition dishes containing moist white-colored filter paper were placed in the adult holding cages for egg deposition. Eggs were air-dried at room temperature for 1–2 days to allow embryonic maturation before being immersed in clean water in individual rearing trays (30 cm (L) × 20 cm (W) × 5 cm (H)). For *Cx. quinquefasciatus*, egg rafts were deposited on free water containers provided to females, followed by transfer using a wooden applicator stick and placed on the water surface in larval trays to allow hatching. Larvae were fed once daily using a commercially sourced protein mixture as larval food (Optimum^TM^ Nishikigoi Carp Fish, Perfect Companion Group Co., Ltd., Samutprakarn, Thailand). Pupae were transferred daily from larval trays to cups containing water and placed directly into steel mesh screen cages (30 cm (L) × 30 cm (W) × 30 cm (H)) for adult emergence.

### 2.2. Insecticide Susceptibility Assays

The procedures for insecticide susceptibility monitoring in adult mosquitoes followed World Health Organization (WHO) standard testing criteria [23] with the recommended discriminating concentrations for susceptibility of 0.25% and 0.75% technical grade permethrin (92.29% purity) for *Ae. aegypti* and *Cx. quinquefasciatus*, respectively. Permethrin was diluted with acetone and silicone oil solution to obtain desired concentration. Individual filter papers (12 cm × 15 cm) were treated using a pipette applying 2 mL of prepared permethrin solution per 180 cm^2^ surface area and air-dried 24 h before use. Control papers were treated similarly with diluent only. Female, 3–5 day-old mosquitoes (nulliparous, non-blood-fed, free-mated) were used in all tests. For each mosquito strain, 25 mosquitoes were exposed in test cylinders for 1 h with either treated or control (without permethrin) papers. Following active ingredient and control exposures, knockdown of mosquitoes at 1 h was recorded for each cylinder, and all mosquitoes were subsequently transferred to separate holding containers and provided 10% sucrose solution. Final knockdown and mortality were recorded at 24 h post-exposure. A total of 100 females (four replicates) of each strain were exposed to permethrin with controls of two replicates (50 females) each. For *Ae. aegypti* (Pu Teuy), the assay used F1 to F3 generation females.

### 2.3. Chemical Attractant

BG-lure^TM^ (Lot number: SC20171, production date: 30 March 2017, Biogents AG, Regensburg, Germany) was purchased from BioQuip^®^ (Rancho Dominquez, Compton, CA, USA). The BG-lure contains a mixture of three active ingredients: 20–<40% of l-(+)-lactic acid (CAS: 79-33-4), 20–<40% of ammonium hydrogen carbonate (CAS: 1066-33-7), and 5–<10% of hexanoic acid (142-62-1) and other inert ingredients.

### 2.4. High-Throughput Screening System (HITSS)

**Figure 1:**
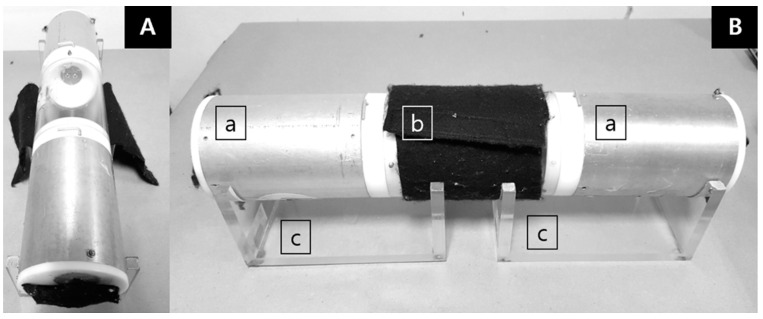
High-throughput screening system (HITSS) for spatial repellency assay (SRA) was applied to evaluate dose–response attraction of BG-lure. (**A**) Front view with uncovered middle cylinder and felt-covered end view window for each chamber. (**B**) Side view with felt-covered middle chamber; a: treated and untreated chambers, b: fabric covered clear cylinder, c: HITSS cradles.

The HITSS device consists of three attached cylinders (Figure 1), which allows several testing options. The HITSS was originally designed to measure toxicity or the behavioral responses of contact excitation and spatial repellency depending on the assay objectives [24]. The middle cylinder (10.2 cm (D) × 15.9 cm (L)) is made of clear acrylic material (Plexiglas^®^) with each end equipped with a butterfly valve opening that controls mosquito movement between cylinders. The middle cylinder has a 1.5 cm opening to allow transfer of the mosquitoes into the cylinder using a mouth aspirator. With values in the open position, mosquitoes can freely access the two adjoining cylinders on either side of the middle cylinder. The two side cylinders are of equal size (10.2 cm (D) × 14.0 cm (L)) and constructed of aluminum. With all three cylinders attached, the total internal volume space is 2.75 L.

The HITSS assay for attractants utilized the spatial repellency design with minor modifications that involved covering the middle chamber and end view windows of each side chambers with dark-colored felt cloth to exclude entry of external light as a potential attractant or repellent for mosquitoes. The attractant assessment was divided into three trials: (1) measuring treatment responses to various doses (from 0.005 g to 10 g) of BG-lure (treatment), (2) 1 g of dry ice as positive control, and (3) one without lure as a negative control. For the treatment HITSS, one side cylinder contained the lure material placed on an aluminum foil dish (3 cm × 3 cm) placed at the far end of the cylinder. The opposite cylinder served as the ‘untreated’ space and was provided with an empty dish only. After lure placement, 20 selected female mosquitoes were released into the middle cylinder and allowed free movement in either direction to determine attraction or repellency. The same procedure applied for both control HITSS setups.

Before testing, female adult mosquitoes were selected on the basis of age and physiological condition—approximately 3–5 days old, nulliparous, and mated. Mosquitoes were provided 10% sucrose solution on a moist cotton wick only and ‘starved’ 12 h before testing (provided water only). For *Ae. aegypti* field mosquitoes (Pu Teuy), F2 to F3 generation females were used. Twenty females were randomly selected using a mouth aspirator and placed in a clean plastic cup with a mesh cover and monitored for 1 h. Only apparent healthy mosquitoes (no evidence of distress or moribund state) were carefully introduced into the central cylinder using an aspirator with the butterfly doors in the closed position. The middle cylinder was covered by a dark fabric to place mosquitoes under dark conditions to avoid possible interference by external laboratory light. The mosquitoes were allowed a 30 s adjustment period inside the holding cylinder before opening the butterfly doors to begin the experiment. The doors remained open for 10 min allowing the mosquitoes to freely move between the three attached cylinders. After 10 min, the doors were closed and the numbers of mosquitoes inside each cylinder were recorded. Each attractant dose was tested using nine replicates (total 180 mosquitoes each dose). To adjust for normal circadian activity, assays for *Ae. aegypti* was conducted during daytime hours (6:00 a.m.–12:00 p.m.) while *Cx. quinquefasciatus* was tested during the first half of the evening (6:00 p.m.–12:00 a.m.).

### 2.5. Analysis

For the WHO susceptibility bioassay, the final mean percentage mortality was adjusted using Abbott’s formula if the control mortality was between 5% and 20% [25]. Findings were interpreted following the WHO criteria [23], wherein resistance is indicated when mortality is below 90%, suspected resistance is indicated if mortality is between 90% and 97% and awaits further testing and confirmation, and susceptibility is indicated if final mortality is between 98% and 100%.

For the HITSS assay, the total numbers of mosquitoes entering the lure-treated (Nt) and untreated (Nu) chambers after 10 min exposure were tabulated. Percentage attraction was calculated using the formula ((Nt − Nu)/(Nt + Nu)) × 100, where 100% represents fully attracted, 0% represents no activity, and −100% represents fully repelled. The Wilcoxon signed rank test was run to compare significance between females in treated or untreated chambers. Results include the mean ± SD (standard deviation) percentage attraction between different amounts of chemical using the Kruskal–Wallis *H* test for multiple comparisons. To observe host-seeking behavior, percentage attraction was compared using a Mann–Whitney *U* test (1) within species: *Ae. aegypti* USDA laboratory strain and Pu Teuy field population, and (2) between laboratory strains: *Ae. aegypti* (USDA) and *Cx. quinquefasciatus* (NIH). All statistical analyses were performed with SPSS version 20 (IBM Corp., Armonk, NY, USA). All tests of significance were set at 5%.

## 3. Results

### 3.1. WHO Bioassay

*Aedes aegypti* (USDA) was completely susceptible to 0.25% permethrin, whereas the recent field-collected Pu Teuy population demonstrated only 6% mortality, indicating very high phenotypic resistance. The *Culex quinquefasciatus* laboratory strain was 100% susceptible to 0.75% permethrin.

### 3.2. BG-Lure Effects

#### 3.2.1. A Pack of BG-Lure (10 g)

**Figure 2:**
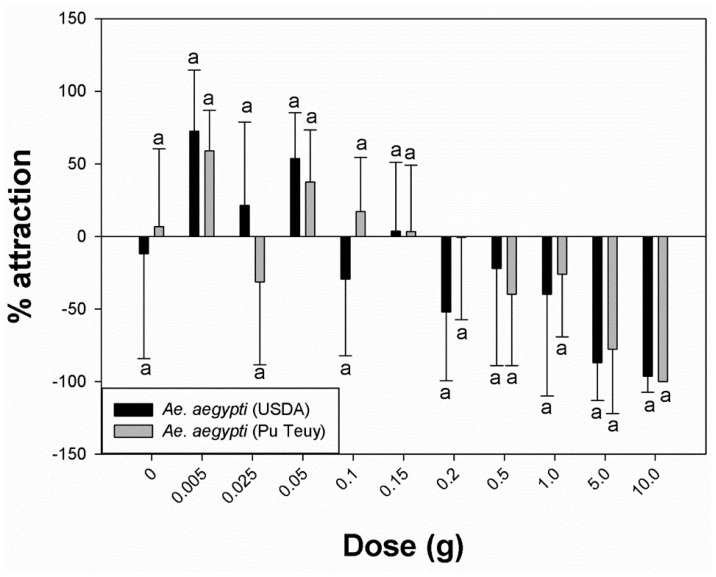
Percentage attraction within species, *Aedes aegypti* USDA (susceptible) and Pu Teuy (resistant) mosquitoes. Different letters between USDA and Pu Teuy indicate statistical significance (Mann–Whitney *U* test, *p* < 0.05).

**Figure 3:**
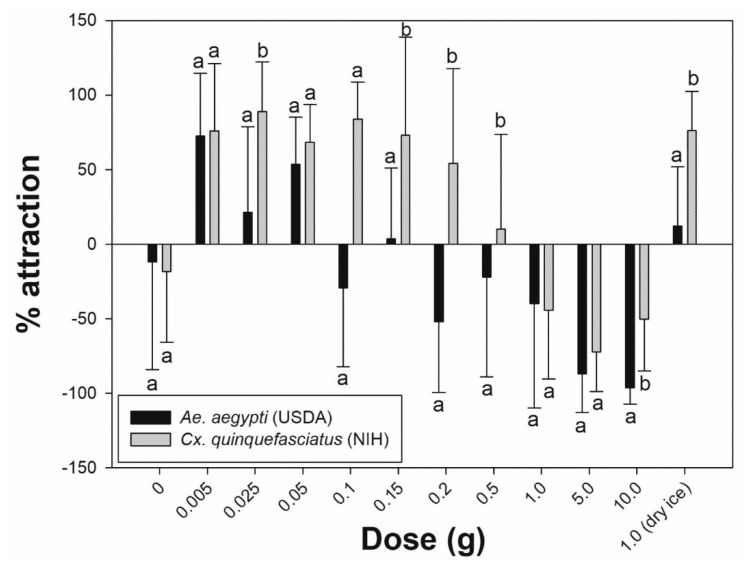
Percentage attraction between pyrethroid susceptible species, *Aedes aegypti* USDA and *Culex quinquefasciatus* NIH. Different letters between species indicate statistical significance (Mann–Whitney *U* test, *p* < 0.05).

Using a complete (single) pack of 10 g commercial lure equally (*p* = 0.317) and strongly repelled permethrin-susceptible (−96.3%) and resistant (−100%) *Ae. aegypti* (Figure 2). The same response was observed for *Cx. quinquefasciatus* (−50.2%), but with significantly less attraction (*p* = 0.002) compared to *Ae. aegypti* (Figure 3). However, when using sequentially smaller doses of chemical, the percentage attraction increased for both species (Figure 3).

#### 3.2.2. Optimizing Dose of BG-Lure

Table 1 shows that the percentage attraction values for *Ae. aegypti* (USDA) exposed to 5.0 g, 1.0 g, and 0.5 g became less repellent as the dose progressively decreased (−87.0%, −40.0%, and −22.2%, respectively). Significantly lower repellency was recorded at 0.15 g compared to 0.2 g (*p* = 0.017). However, between 0.2 g and 0.1 g, the percentage attraction fluctuated from −51.9% (0.2 g) to 3.5% (0.15 g) and −29.3% (0.1 g). A further reduction in dose weight (0.05 g, 0.025 g, and 0.005 g) maintained clear positive values (53.5%, 21.3%, and 72.6%, respectively), indicating attraction. Even though 0.05 g showed a lower value in percentage attraction compare to 0.005 g, it was not statistically significant (*p* = 0.287). Furthermore, the mean ± standard deviation number of mosquitoes attracted to the treated cylinder was greatest at 0.05 g (untreated: 6.4 ± 2.7 vs. treated: 1.9 ± 1.5), and the amount induced the highest response rate. These inverse patterns of response showing increased attraction with reduction in dose were similar in the *Ae. aegypti* field population (Figure 2). Overall, there were no significant differences in percentage attraction responses between the permethrin-susceptible laboratory strain and the resistant field population (Figure 2). *Culex quinquefasciatus* showed positive percentage attraction values to a wider range of doses that was significantly different from *Ae. aegypti* (Figure 3). *Culex* females began showing attraction at 0.5 g (10.0%) and continued with positive attraction values down to 0.005 g (75.9%). At 0.025 g, mosquitoes produced the strongest attraction response (88.9%), with a mean number of females in the treated cylinder at 6.0 ± 3.7.

#### 3.2.3. Dry Ice (1 g)

Different responses were detected between *Ae. aegypti* (USDA) and *Cx. quinquefasciatus* (NIH) using the dry ice ‘positive’ control (Figure 3). One gram of dry ice strongly attracted *Cx. quinquefasciatus* with 76.0% ± 26.3% attraction (10.1% ± 2.8% in treated cylinder vs. 1.6% ± 1.8% in untreated) with a response rate greater than all other chemical doses. *Ae. aegypti* (USDA) had a significantly reduced (*p* = 0.002) percentage attraction to carbon dioxide (12.2%), equal to 0.15 g of BG-lure (3.5%), and it was only exceeded by 0.05 g (53.5%), 0.025 g (21.3%), and 0.005 g (72.6%) doses.

## 4. Discussion

This study demonstrates that the HITSS assay is a simple and acceptable test system to screen and evaluate potential chemical attractants. Furthermore, compounds can be ‘optimized’ by dose for attraction in a small operating space (2.75 L volume for three-cylinder configuration), which might provide useful indications of scaling up to higher doses required for larger spaces. Overall findings show the amount of BG-lure was successfully evaluated using the HITSS assay, suggesting that other chemical compounds should be amenable to screening using this method. Secondly, clear differences in response between the two mosquito species was observed suggesting differing activation thresholds of olfactory receptors for each species using the commercial attractant blend. This indicates that species-specific attractants (or dosage) might be considered when optimizing trapping systems for target insects in the field [26]. Thirdly, there was no difference in dose responses to the chemical between a permethrin susceptible laboratory strain and a highly resistant field-derived population of *Ae. aegypti*. Lastly, dose–response measures clearly showed opposing actions of the compound depending on the dosage used. For stimulating attraction, lower doses were required, while repellency was incited at the higher dose range.

Various mechanical and passive trapping devices of mosquitoes have been developed for research, operational monitoring, and/or control purposes (removal trapping) [27]. To enhance capture efficiency, a mosquito trap typically might include one or more olfactory or visual attractants to draw mosquitoes to the trap [28,29,30,31,32]. In the laboratory, olfactometers are commonly used for evaluating potential lure candidates, and this can provide useful information before conducting a larger scaled semi-field or field trial. As the laboratory-sized preliminary tests are not always applicable in real situations, it is crucial to perform the next level of field trials. On the basis of the field test results, the accuracy of the laboratory sized olfactometers can be evaluated [33,34,35,36,37,38]. Tests using wind tunnels represent another option to observe insect response to attractants [39]. However, these devices and setups require precise operational conditions (e.g., airflows and filter systems). Therefore, they are of limited use except in more sophisticated laboratories.

This study represents an investigation for measuring the dose response of mosquitoes to an attractant compound using a simple, horizontal passive device without the requirement of mechanical airflow (see vertical passive diffusion assay [40]). The HITSS is a versatile device that allows, depending on test design configuration, the ability to measure four actions: toxicity, spatial repellency, contact repellency, and attractive properties of chemicals. The HITSS device was originally designed for screening toxic and repellent properties [24,37]. This study demonstrates that, with only a few minor modifications, the HITSS is adaptable as a laboratory-based assessment for evaluating attractants.

The commercialized BG-lure was designed for enhancing sampling and monitoring of mosquitoes in surveillance programs, as well as providing some level of adult control. The particular formulation and complementary trapping system incorporating the lure was initially designed to focus on day-active *Aedes* mosquitoes. Interestingly, use of the HITSS assay showed *Cx. quinquefasciatus*, a typically night-active species, to have a much higher degree of attractiveness and over a wider range of dose than either of the *Ae. aegypti* strains used in this study. However, the *Culex* used is a long-adapted insectary strain that merits caution when extrapolating these behavioral findings to natural populations.

The commercial lure used in this study, a blend of several known bioactive components (lactic acid, ammonia, and hexanoic acid), successfully attracted female *Ae. aegypti* and *Cx. quinquefasciatus*. Mosquitoes possess different chemoreceptors with primary receptor neurons mostly associated with the antennae and maxillary palps [12,41,42]. Presumably, each species have different numbers and specific kinds of olfactory receptors between them [43]; thus, observing varying responses to lure and dry ice between two phylogenetically distance species with very different bionomics and ecologies is not an unexpected finding.

This study used CO_2_ (alone) as the ‘positive’ control attractant as comparison. Under HITSS assay conditions, the sublimated CO_2_ released from 1 g of dry ice was sufficient to strongly attract *Cx. quinquefasciatus* (76.0% attraction), but significantly less so with *Ae. aegypti* (12.2% attraction, *p* = 0.002). Carbon dioxide is a powerful neural activator for upwind orientation (positive anemotaxis) of most mosquito species toward vertebrate hosts [3,11]. Both *Ae. aegypti* and *Cx. quinquefasciatus* are no exception [44,45,46,47]. Carbon dioxide is a potent activator of female *Ae. aegypti* even at very low concentrations (10 ppm or >0.04% above ambient atmospheric levels) [48], evoking oriented flight along the plume stream, resulting in rapid source finding [49]. However, at higher concentrations, CO_2_ can repel mosquitoes [7]. For example, capture of *Cx. quinquefasciatus* decreased significantly when the CO_2_ release rate increased from 300 to 1000 mL/min [50]. A negative chemotropism was observed with this species as CO_2_ concentrations increased [51].

For both species, CO_2_ appears to have a synergistic action with host odors in the attraction of female mosquitoes [33,47]. However, the presence of other host odors appears to supersede CO_2_ in the induction of orientation. Under field conditions, the combination of BG-lure and CO_2_ applied to traps showed that the latter was the predominant attractant cue for trapping *Aedes albopictus* (Skuse) [52,53]. In this study, no competing kairomones (attractants/stimulants) were present with CO_2_. Many mosquito traps use this attractant to increase mosquito captures with or without other synthetic odorants and visual cues [27]. However, the influence of environmental conditions such as heat and humidity in combination with CO_2_ generates a greater number of female *Ae. aegypti* compared to either carbon dioxide with heat or moisture alone [54]. In the HITSS study, only background laboratory temperatures and relative humidity were present during testing.

The amount (dose) and proportion of chemical mixtures in compounds can play critical roles in insect response and are important considerations in attractant development [55]. As the commercial lure combination of ingredients (by proportion) was not modified in this study, it was evident that dose alone had a significant effect on responses between the two species. The HITSS assay identified 0.005 g and 0.025 g of lure producing the peak attraction response in *Ae. aegypti* and *Cx. quinquefasciatus*, respectively. The positive percent attraction for *Cx. quinquefasciatus* included a greater range of dosing (0.5 g to 0.005 g) compared to *Ae. aegypti* USDA (0.05 g to 0.005 g) and Pu Teuy (0.15 g to 0.005 g). Alternatively, as dosing gradually increased to 10 g, females showed repellency. These dose-dependent reversal responses were reported by previous studies [56,57] as common responses, and it was confirmed in a recent study using plant volatiles that lower doses attracted while higher doses caused an avoidance response [58,59].

Carbon dioxide is widely recognized as the most ideal and universal mosquito attractant; however, its availability is often limited logistically and can be operationally costly. Although other methods for generating CO_2_ have been devised and shown to also attract mosquitoes, both convenience and cost can be factors depending on the system used (e.g., byproduct of yeast and sugar fermentation or propane combustion) [60,61,62]. These obstacles have been one of the prime motivations for seeking alternative synthetic chemical attractants to obviate the need for CO_2_. The many compounds evaluated and used, generally as mixtures or in combination with other attractants such as CO_2_, include l-lactic acid, butanone, ammonia, isovaleric acid, and 1-octen-3-ol [14,15,17,34,35,46,63]; however, effective ratios between these compounds must be established to achieve maximum synergistic effects. The HITSS may provide an acceptable addition or alternative assay to other attractant-based technologies for study on these and other chemical combinations.

Various types of measurement tools have been used to evaluate chemical lure candidates [35,39,48]. Most devices generate artificial air currents that carry a chemical plume that requires a mosquito to fly upwind to the source. However, the air flow itself also can attract mosquitoes [64,65], thus potentially confounding results. Some olfactometers without use of air movement have shown that mosquitoes can detect and direct flight movement to the chemical source in a passive system [3,39]. Similarly, the HITSS assay is a passive system without the use of directed air flow. Another advantage is the compact size of the HITSS assay making it easy to set up and use compared to many olfactometers which are large and complicated devices designed to provide air flow and highly regulated chemical concentration discharge. However, the smaller working volume (2.75 L) between the three HITSS cylinders may also present limitations, resulting in inconclusive results either by chance through flight movement alone or external background factors that influence behavior. The use of adequate controls and increasing test replicates can help to alleviate some of these potential problems. Another potential limitation to this test design is the use of a short period of exposure (10 min); thus, the low overall response rate reported using the commercial lure may reflect insufficient time for a mosquito to fully react to a chemical. Further experimentation is required to examine responses over a longer period of exposure. However, within the enclosed HITSS system, each cylinder can maintain different gradations from initial input (e.g., treatment vs. none) for a while (ca. 10 min) before the atmospheric components in all cylinders become more equilibrated [24].

Unlike the typical HITSS setup [24], in this study, the covering of the view windows at each end of the side cylinders may have resulted in a lower response rate of *Ae. aegypti* by eliminating potential phototaxic-directed movement. The normally nocturnal *Cx. quinquefasciatus* had overall higher responses than the diurnal *Ae. aegypti* at all data points (Figure 2). These results may reflect the differences between nocturnal and diurnal active species under dark test conditions. This is indicated in the study findings for both *Ae. aegypti* and *Cx. quinquefasciatus* depending on the dose used (Figure 3). 

The HITSS assay could successfully optimize the amount of BG-lure for attraction. However, either above or below the optimized dose ranges, the attraction level decreased dramatically. In other words, the pattern of attraction is not in a direct proportion ratio as described in Figure 2. The attraction thresholds for each species, especially the starting points of repellency, are shown in Figure 2 and Figure 3. Ideally, establishing attractant con-centration ranges between the peak point and the threshold (just before repellency begins) for each species could be a standard for rapid screening and evaluating multiple lure candidates. Currently, the typical HITSS is not designed to sample and measure concentrations of component molecules inside the cylinders. Having this added capacity would lend a great deal more potential for screening purposes.

When the HITSS assay indicates high preference indices, caution should be exercised as this may not reflect how a chemical may perform in other testing designs, especially those using much larger volumes of space to interact with mosquitoes and the surrounding environment. This study represents the first attempt to evaluate a chemical lure using the HITSS assay, and the first in a series of studies looking at other candidate compounds to provide initial indications for identifying potential chemical lures before moving on to further development phases using other laboratory- and field-based methods.

In conclusion, allowing free movement between test cylinders in a noncompetitive study design, the HITSS assay appears suitable for rapidly screening chemical actions of attraction (and repellency thresholds) of different chemicals. However, with some inherent limitations to the procedure, the use of various olfactometer and wind-tunnel experiments would augment response findings derived from the HITSS assay. Further studies using the HITSS to evaluate other potential attractants and various chemical compound mixtures on different species under varying physiological conditions (e.g., age, parity) are warranted. Most importantly, when acceptable candidate compounds are identified using this laboratory-based assay, they should be evaluated in a stepwise progression under semi-field (e.g., screen-enclosed facility) and natural field settings to optimize trapping systems. Future studies using this simple assay system for identifying action profiles of bioactive volatile compounds as potential attractants should contribute to an accelerated development of effective mosquito trapping tools.

## 5. Conclusions

We evaluated a simple olfactometer without the need of airflow, i.e., the high-throughput screening system (HITSS). The HITSS device successfully optimized a commercial mosquito attractant, Biogents-lure (BG-lure^TM^). Results showed dose-dependent responses with 0.005 g leading to the highest attraction for *Ae. aegypti*, while doses of 0.2 g and above produced a repellent response. There was no significantly different response behavior between permethrin-susceptible and -resistant *Ae. aegypti*. *Cx. quinquefasciatus* showed significantly different responses compared to *Ae. aegypti* by producing attraction over four times a wider range of amounts. These results demonstrate a simple olfactometer device to screen potential chemical attractants without use of an air-plume, thus expanding testing capabilities beyond more sophisticated laboratory settings.

## Figures and Tables

**Table 1:** Mean ± SD percentage attraction of *Aedes aegypti* and *Culex quinquefasciatus* for different doses of BG-lure.

Species	Amount (g)	Mean ± SD No. of Females ^†^ in HITSS Chambers		*p*-Value ^†^	Mean ± SD Percent Attraction ^††^
		Untreated	Treated		
*Ae. aegypti* (USDA)	0.0	2.2 ± 1.7	1.3 ± 1.1	0.203	−11.9 ± 72.2d
	0.005	0.6 ± 0.9	3.0 ± 2.1	0.011 *	72.6 ± 42.0a
	0.025	2.0 ± 1.6	2.2 ± 1.3	0.670	21.3 ± 57.4b
	0.05	1.9 ± 1.5	6.4 ± 2.7	0.007 *	53.5 ± 31.8ab
	0.1	1.8 ± 1.1	1.1 ± 1.1	0.132	−29.3 ± 52.9d
	0.15	2.1 ± 0.9	2.6 ± 1.7	0.389	3.5 ± 47.4c
	0.2	1.0 ± 0.9	0.2 ± 0.4	0.020	−51.9 ± 47.5e
	0.5	0.8 ± 1.4	0.2 ± 0.7	0.357	−22.2 ± 66.7d
	1.0	1.1 ± 1.5	0.2 ± 0.4	0.084	−40.0 ± 70.0de
	5.0	3.0 ± 1.0	0.2 ± 0.4	0.007	−87.0 ± 26.1f
	10.0	5.7 ± 3.5	0.1 ± 0.3	0.007	−96.3 ± 11.1f
	1.0 (dry ice)	1.7 ± 0.9	2.6 ± 1.9	0.206	12.2 ± 39.6c
*Ae. aegypti* (Pu Teuy)	0.0	2.1 ± 1.2	2.0 ± 1.1	0.943	1.1 ± 56.9c
	0.005	1.0 ± 0.9	4.2 ± 1.8	0.007 *	58.9 ± 27.9a
	0.025	1.3 ± 1.3	0.7 ± 0.9	0.236	−31.5 ± 56.8d
	0.05	1.4 ± 0.7	3.2 ± 1.2	0.014 *	37.4 ± 35.9b
	0.1	1.6 ± 1.3	1.8 ± 1.0	0.480	17.0 ± 37.3c
	0.15	2.7 ± 1.7	2.9 ± 2.0	0.621	3.3 ± 45.8c
	0.2	1.9 ± 1.5	1.8 ± 1.2	0.994	−2.2 ± 61.8d
	0.5	3.7 ± 2.2	0.4 ± 0.7	0.011	−82.0 ± 33.9e
	1.0	1.3 ± 1.4	0.2 ± 0.4	0.039	−48.1 ± 50.3d
	5.0	1.7 ± 1.1	0.1 ± 0.3	0.016	−77.8 ± 44.1e
	10.0	5.4 ± 2.2	0.0 ± 0.0	0.008	−100.0 ± 0.0f
	1.0 (dry ice)	N/A	N/A	N/A	N/A
*Cx. quinquefasciatus* (NIH)	0.0	3.6 ± 2.7	3.7 ± 3.2	1.000	−18.4 ± 47.4d
	0.005	0.4 ± 0.7	4.9 ± 3.3	0.011 *	75.9 ± 45.0b
	0.025	0.2 ± 0.7	6.0 ± 3.7	0.012 *	88.9 ± 33.3a
	0.05	1.1 ± 1.1	5.4 ± 2.2	0.007 *	68.3 ± 25.4c
	0.1	0.6 ± 0.9	4.8 ± 2.2	0.008 *	83.7 ± 24.9b
	0.15	0.4 ± 0.7	3.9 ± 3.4	0.028 *	73.1 ± 65.6b
	0.2	0.9 ± 1.2	3.2 ± 2.5	0.068	54.2 ± 63.5c
	0.5	1.2 ± 1.1	1.3 ± 1.1	0.915	10.0 ± 63.6d
	1.0	4.1 ± 2.1	1.9 ± 1.5	0.024	−44.4 ± 46.1e
	5.0	4.1 ± 2.4	1.0 ± 1.2	0.007	−72.2 ± 26.8f
	10.0	4.6 ± 3.1	1.2 ± 0.8	0.018	−50.2 ± 34.7e
	1.0 (dry ice)	1.6 ± 1.8	10.1 ± 2.8	0.008 *	76.0 ± 26.3b

Nine replicates each dose, 20 females each replicate (180 total). ^†^ Compared no. of females in untreated and treated chambers using Wilcoxon signed rank test (*p* < 0.05). * Significantly more females attracted in treated chamber compare to untreated chamber (*p* < 0.05). ^††^ Percentage attraction = (# females in treated − untreated)/(# females in treated + untreated) × 100. Different letters within a column indicate significant differences between species-specific doses by Kruskal–Wallis *H* test for multiple comparisons (*p* < 0.05).

## Data Availability

The datasets supporting the conclusions of this article are included within the article. Raw data are available from the corresponding author on reasonable request.
